# An evaluation of adherence to ethical codes among nurses and nursing students

**Published:** 2017-07-05

**Authors:** Mostafa Bijani, Fariba Ghodsbin, Sorur Javanmardi Fard, Fateme Shirazi, Farkhonde Sharif, Banafsheh Tehranineshat

**Affiliations:** 1 *PhD Candidate in Nursing, Student Research Committee, Shiraz University of Medical Sciences, Shiraz, Iran.*; 2 *PhD Candidate in Nursing, Student Research Committee, Quality Improvement in Clinical Education Research Center, School of Nursing and Midwifery, Shiraz University of Medical Sciences, Shiraz, Iran. *; 3 *Department of Community Health Nursing, School of Nursing and Midwifery, Shiraz University of Medical Sciences, Shiraz, Iran.*; 4 *Student Research Committee, Shiraz University of Medical Sciences, Shiraz, Iran. *; 5 *Professor, Community Based Psychiatric Care Research Center, School of Nursing and Midwifery, Shiraz University of Medical Sciences, Shiraz, Iran.*

**Keywords:** *Adherence*, *Ethical codes*, *Nursing students*, *Registered nurse*, *Nursing ethics*

## Abstract

Ethical codes are instructions that shape ethical behavior and determine which values and beliefs should be accepted. These codes act as a practical guideline in the nursing profession. The present study aimed to compare adherence to ethical codes between the nursing students and working nurses of Valiasr Hospital, affiliated with Fasa University of Medical Sciences in Fars Province, Iran. In this descriptive-analytical study, the data collection tool was nurses’ self-reporting questionnaire on adherence to ethical codes devised by Mahdavi Lenji and Ghaedi Heidari, who have also confirmed its validity and reliability. This questionnaire consisted of 3 sections: personal information, ethical codes related to clinical service provision (23 items), and relationship with the treatment team (8 items). A total of 400 individuals (178 nursing students and 222 nurses) were selected through census sampling method**.** Data were analyzed through descriptive, ANOVA and t-test statistical methods using SPSS 22 software. There was no significant difference between nurses and nursing students in terms of adherence to ethical codes related to clinical service provision, but the latter achieved a significantly higher score in codes on relationship with the treatment team (*P *= 0.04). Although the score of nursing students was higher than nurses in comparison to the treatment team, they lacked the necessary technical competency to adhere to ethical codes. Therefore, we recommend that nursing instructors and educational managers pay more attention to teaching nursing ethics and supervise their implementation and practicality in clinical environments.

## Introduction

In recent years, healthcare communities have decided to formulate their norms and values as codes of ethics (1). Considering cultural differences, ethical values in nursing and healthcare as a human-oriented system have attracted a lot of attention (2). Advancements in technology, increased costs of healthcare, and organizational changes in the society and the healthcare system have created significant problems and challenges for nurses. As a result, they need professional competence and a framework provided by code of ethics to make correct judgments during work, and that is why the development of ethical codes is considered so vital (3, 4).

Ethical issues are rather frequent in the nursing profession, and in order to resolve complicated ethical problems, we need a basic theoretical understanding of ethical decision-making (5). Designing a nursing code of ethics is a way to express shared values and responsibilities specific to this profession (6). These values guide the decisions and actions of nurses and provide information to the nursing community throughout the world (4). 

Ethical codes are principles and value systems in a profession based on which the ethical behavior of its members is evaluated (7). The code of professional ethics for nurses was first designed by the International Council of Nurses (ICN) in 1953. This code specified the nurses’ professional responsibilities toward patients, the nursing profession, coworkers, other professional healthcare team members, and the society (3).

In Iran, there have been several attempts to design a code of ethics in medical fields, with an emphasis on religious values and their integration into these codes (8). The Ministry of Health and Medical Education (MOHME) established the Medical Ethics Research Center in 1993 and formed medical ethics research committees on the national level in 1997 (9). Another important measure in this regard was designing the National Code of Ethics delivered in early 2011(10) for professional nurses working in educational, research, management, health, and clinical sectors at all levels. This code has been revised and updated when necessary (11). 

Studies have shown that knowledge and application of ethical codes is not so practical for nurses (12). Many nurses do consider the code of professional ethics when faced with ethical issues, but do not act upon it. In most cases, they do not possess the power and support to react according to the code, and sometimes they simply do not know what to do (13). 

A few studies have been conducted on the level of adherence to ethical codes in nursing students and nurses, but the results are contradictory. For instance, according to one study, 70% of patients, 86% of nurses and 53% of nurse managers described nurses' adherence to ethical codes as satisfactory (14). Yet another study showed that only 41.8% of patients found nurses' adherence to ethical codes to be satisfactory (15). Joolaee et al. stated that only 39.7% of patients were highly satisfied with nursing services (16). According to Borhani et al., although most nurses and nursing students were aware of nursing ethics and its significance in their profession, they were highly concerned about their own capability to implement ethical codes in clinical settings (17).

If we can train strong nurses who are capable of providing comprehensive and holistic care, the nursing profession will improve. Consequently, the nurse's influence on the healthcare system will increase, which can result in higher satisfaction of healthcare beneficiaries (18). 

Nursing students constantly attend educational hospitals and encounter ethical issues involving nurses and patients (15). Therefore, an appropriate plan to improve ethical performance, clinical decision-making and nursing services should be based on the required ethics and training needs. Hence, it is vital to evaluate adherence to ethical codes among nursing students as the future employees of the healthcare system, and nurses as individuals offering healthcare services. The majority of the qualitative studies on nursing ethics conducted in Iran did not investigate adherence to ethical codes and the effect on nursing students and nurses. Thus, in order to provide a background and predict the performance of future nurses in terms of ethical codes, enhance the quality of clinical decision-making, offer safety care, and draw a comparison between the current and future nurses, this study was conducted using a quantitative method. The study aimed to compare adherence to the code of ethics among the nursing students and nurses in Valiasr Hospital, affiliated with Fasa University of Medical Sciences in Fars Province, Iran.

## Method

This was a descriptive-analytical study designed to compare and evaluate observance of ethical codes among the nursing students and working nurses of Valiasr Hospital, affiliated with Fasa University of Medical Sciences. Research population consisted of full-time working nurses holding a bachelor’s degree or higher, as well as nursing students in their junior and senior years. Sampling was done via non-random census method across all Valiasr Hospital wards, except the VIP (very important person) wards. As a result, 400 individuals (178 nursing students and 222 nurses) were selected to participate in the study. Inclusion criteria for the nursing students were: enrollment in a bachlor’s program, not being a guest or transfer student, and having no prior work experince in official healthcare centers. Inclusion criteria for the nurses were: a bachelor’s or higher degree, and a minimum of one month’s experience as a professional nurse in different wards. Researchers went to all wards in three working shifts from May to Auguest 2016. They stated the aim of the study and collected data after obtaining a written informed consent form all participants. Data were collected from the participants by a questionnaire consisting of 3 parts. In view of ethical considerations, permission was obtained from hospital management, and confidentiality of information was assured. The nurses' self-reporting questionnaire on adherence to ethical codes was used as the data collection tool, which was devised by Mahdavi Lenji and Ghaedi Heidari (8) based on the code of ethics published by the Ministry of Health and Medical Education (19). The questionnaire consisted of 3 sections: 1) demographic information such as age, gender, work experience, education and academic semester; 2) questions on ethical codes related to clinical service provision (23 items); and 3) codes related to the treatment team (8 items). Responses were graded based on a five-degree Likert scale (5: always, 4: often, 3: sometimes, 2: rarely, and 1: never). The minimum and maximum total scores were 23 and 115 for the second section, and 8 and 40 for the third section, respectively. The questionnaire was given to 7 nursing and medical experts in the field of professional ethics to confirm its content and face validity. The questionnaire was given to 15 nursing students and 15 nurses in order to verify its reliability, which was then calculated by Cronbach’s alpha to be 0.90% (8). Data were analyzed through inferential and descriptive statistical methods (ANOVA and t-test) using SPSS 22. This study was approved by the Research Council and the Ethics Committee of Fasa University of Medical Sciences, Fars province, Iran (Code: EC-95090).

## Results

Our findings showed that of the 400 subjects, 222 (55.5%) were nurses and 178 (44.5%) were nursing students. There were 145 men and 77 women among the nurses, with an average age of 31.36 ± 1.07, of which 210 had a bachelor’s degree in nursing and 12 had a master’s degree. Moreover, 109 nursing students were men and 69 were women, with an average age of 22.83 ± 3.52. 

In the section related to clinical service provision, average total scores were 85.75 ± 13.20 and 84.52 ± 17.39 for nursing students and working nurses, respectively. The t-test did not show any significant difference between nurses and nursing students in this area. However, in the section related to relationship with the treatment team, average total scores of nursing students and working nurses were 30.18 ± 5.03 and 29.01 ± 6.23, respectively, and the t-test revealed a significant difference between the two groups (*P *= 0.04 and t = 2.04) (Table 1).

**Table 1:** Comparison between the average scores for adherence to ethical codes among nursing students and nurses

Adherence to ethical codes	Group	Mean ± SD	t-test
Clinical service provision	Nursing students	85.75 ± 13.20	t = 0.77 *P *= 0.42
	Nurses	84.52 ± 17.39	
Relationship with the treatment team	Nursing students	30.18 ± 5.03	t = 2.04 *P *= 0.04
	Nurses	29.01 ± 6.23	

*P* value < 0.05 was considered significant. *SD: Standard Deviation*

In the section on ethical codes related to clinical service provision, average total scores were 86.70 ± 16.03 and 82.22 ± 14.52 for women and men, respectively. The t-test revealed significant differences in this area (*P *= 0.006, t = 2.76). As regards the relationship with the treatment team, average total scores were 30.28 ± 5.77 and 28.23 ± 5.49 for women and men, respectively. Moreover, there was a significant difference between the two groups according to t-test results (*P *= 0.01, t = 3.45) (Table 2).

**Table 2:** Comparison between the average scores for adherence to ethical codes among nursing students and nurses based on gender

Adherence to ethical codes	Group	Mean ± SD	t-test
Clinical service provision	Female	86.70 ± 16.03	t = 2.76 *P *= 0.006
	Male	82.22 ± 14.52	
Relationship with the treatment team	Female	30.28 ± 5.77	t = 3.45 *P *= 0.001
	Male	28.23 ± 5.49	

*P* value < 0.05 was considered significant. *SD: Standard Deviation*

The findings showed that the highest average scores for adherence to ethical codes pertained to "respecting the client/patients’ privacy when performing nursing interventions", which were 4.20±0.88 and 3.96 ± 1.01 for nursing students and nurses, respectively. Additionally, the lowest average score of adherence to ethical codes pertained to "introducing themselves and stating their title and professional role" (3.15 ± 1.16) in nursing students. For nurses, the lowest score belonged to "consulting the hospital ethics committee about decision-making when confronting ethical challenges" (3.09 ± 1.03) (Table 3).

**Table 3:** The average scores for adherence to ethical codes in nursing students and nurses

**Items **	**Nursing students**	**Nurses**
	Mean ± SD	**Mean ± SD**
Introducing themselves and stating their title and professional role	3.15 ± 1.16	3.45 ± 0.85
Providing all nursing interventions while respecting the clients/patients and preserving their dignity	3.98 ± 0.82	3.85 ± 0.94
Considering the clients/patients’ demands regardless of their age, sex, race, economic status, lifestyle, culture, religion, political beliefs and physical abilities	3.93 ± 0.88	3.91 ± 0.92
Performing nursing care based on current knowledge and common sense	3.85 ± 0.81	3.84 ± 0.93
Establishing gentle behavioral and verbal communication with the clients/patients in a way that wins their trust, and attempting to understand their needs and concerns	4.06 ± 0.80	3.82 ± 0.97
Obtaining the patients’ or their legal guardians’ consent before performing any nursing interventions, and providing sufficient information in the process	3.42 ± 0.96	3.56 ± 0.93
Having a complete knowledge of the risks when presenting or applying a new product in clinical practice, and providing the client/patient with the necessary information about the benefits and disadvantages of the product to promote informed decision-making	3.29 ± 0.89	3.59 ± 0.95
Being aware that no one has the right to consent in place of a competent adult. In case of children, giving the consent is one of the legal guardian’s responsibilities.	3.85 ± 0.71	3.73 ± 0.98
Educating the clients/patients and their families in order to empower them within the framework of their specific care plan and discharge program	3.26 ± 1.06	3.47 ± 0.95
Starting the necessary intervention without the client/patient’s consent only as an exception and in case of an emergency, when immediate therapeutic action is required to save the client/patient’s life	3.77 ± 0.79	3.67 ± 0.99
Performing the appropriate intervention based on existing standards and client/patient’s higher interests when obtaining the informed consent or realizing the client/patient’s consent or wishes is not possible	3.69 ± 0.75	3.51 ± 1.01
Applying safety measures to ensure that nursing interventions are harmless, and when necessary, consulting other health team members	3.86 ± 0.82	3.58 ± 1.08
Considering all the information given or obtained during the care process as professional secrets, and not revealing them without the client/patient’s approval except in legally permissible cases	3.89 ± 0.83	3.56 ± 1.15
Employing the medical information of clients/patients only for health-related purposes (such as treatment or research) and in the patients’ interest, and informing the clients/patients that part of their medical record might be disclosed to other team members for medical consultation	3.88 ± 0.88	3.49 ± 1.09
Using the client/patient’s medical information in research or education only with their permission, and presenting the results without mentioning the name, address or any other information that could lead to identification of the patient	3.65 ± 0.95	3.31 ± 1.20
Respecting the client/patient’s privacy when performing nursing interventions	4.20 ± 0.88	3.96 ± 1.01
Respecting the patient’s bill of rights	3.94 ± 0.80	3.80 ± 0.96
Providing care for injured individuals or clients/patients in emergency situations, even outside the workplace	3.52 ± 0.98	3.66 ± 0.84
Respecting clients/patients’ right to change the charged nurse or other healthcare providers in case of dissatisfaction or any other problem, and trying to satisfy the clients/ patients in this matter as much as possible	3.80 ± 0.92	3.73 ± 0.90
Informing the authorities who have sufficient power to improve the conditions in case of noticing a violation of standards of care	3.73 ± 0.89	3.82 ± 0.88
Reporting any complaints or problems of the clients/patients to the ward supervisor	3.71 ± 0.98	3.68 ± 0.86
Avoiding any action, even by the client/patient’s request, that requires ethical, legal or religious violation	3.96 ± 0.86	3.89 ± 0.96
Assisting the clients/patients who spend the last days of their lives to accept the reality and to appropriately plan their demands, including performing religious practices or recording their wills	3.31 ± 1.17	3.63 ± 0.82
Cooperating with medical team members, the clients/patients and their families to offer nursing interventions more efficiently	3.75 ± 0.88	3.82 ± 0.88
Helping the clients/patients or their legal guardians make decisions about nursing interventions	3.56 ± 0.87	3.69 ± 0.87
Sharing one’s professional knowledge and experience with other colleagues	3.74 ± 0.94	3.73 ± 0.89
Establishing a good relationship based on mutual trust with the medical team	3.89 ± 0.82	3.75 ± 0.93
Having a respectful conduct toward other nurses, professors and students	0.86	3.76 ± 1.01
Discussing any conflicts of interest in the care of clients/patients with senior colleagues and principals, and giving priority to preserving the clients/patients’ rights	3.93 ± 0.76	3.58 ± 1.00
Establishing a respectful professional relationship with co- workers in various levels, including head nurses, supervisors, nursing directors and department heads	3.95 ± 0.81	3.60 ± 1.01
Consulting the hospital ethics committee about decision- making when confronting ethical challenges	3.27 ± 1.16	3.09 ± 1.03

*SD: Standard Deviation*

The ANOVA test did not reveal significant differences between the nurses’ education and work experience and the ethical codes related to clinical service provision or relationship with the treatment team. Likewise, there was no relationship between the students’ semester and the ethical codes in the domains of clinical service provision and relationship with the treatment team.

## Discussion

Findings of this study showed that there were no significant differences between the average scores of nursing students and professional nurses regarding their adherence to ethical codes related to clinical service provision. However, a significant difference was observed in the section on codes for relationship with the treatment team, denoting that the nursing students adhered to these ethical codes more than the working nurses. This could be due to the students’ academic training at the university and during training workshops on ethics. It is worth mentioning that unlike the students, nurses were faced with a heavy workload, low nurse to patient ratio, and lack of time. In Mahdavi Lenji and Ghaedi Heidari’s study in Iran, there were no significant differences between the performance of students and nurses regarding codes related to clinical service provision, while nurses had better adherence levels in terms of codes pertaining to relationship with the treatment team (8). Erdil and Korkmaz believed that since nurses are faced with ethical problems in their work, they have to have the ability to identify them, and this can only happen when nurses learn the theoretical concepts of ethics during their academic education (20). Verpeet et al. found that an ethical code could serve several functions, for instance support nurses’ professional identity, and provide directions for nursing practice. Moreover, they expressed viewpoints on concept, including an investigation of nurses' duties in a communicative one, and proposed that particular attention should be paid to the personality of nurses and to the nature of nursing practice as a communicative activity. Verpeet et al. insisted that we need to develop effective and practical tools to enhance nursing ethics, for instance by developing workable methods and educational courses. Such measures will empower nurses and encourage them to provide input as they reflect on the ethical issues that are dealt with in the code, and help them utilize the code in their practice (4). In this regard, Hartrick Doane stated that we need to focus on nursing teachers and cooperation among clinical training institutions, staff, and most importantly students, and these trainings need to be continuous and based on organizational and environmental factors (21). 

In this study, the highest average scores for adherence to ethical codes among both nursing students and nurses pertained to "respecting the client/patients’ privacy when performing nursing interventions”. The lowest average scores pertained to "introducing themselves and stating their title and professional role” in nursing students, and “consulting the hospital ethics committee about decision-making” when confronting ethical challenges" in nurses.

In a Dobrowolska et al. study on assessment and comparison of International Council of Nurses (ICN) codes of ethics in UK, Ireland, Holland, and Poland, findings indicated that in all those countries, respect for patients had the highest priority for nurses in their professional practice. In the present study, both groups achieved high scores with regard to respect for patients as well, which is demonstrated in the section related to clinical service provision (22). Mohajjel Aghdam et al. found that from the patients’ perspective, nurses’ highest performance pertained to "respectful behavior toward their colleagues, superiors and nursing students" (45.8%), and their lowest performance was related to “introducing themselves to patients by announcing their names, roles and job titles" (9.8%) (15). In a study by Khaki et al., the highest average scores for adherence to professional ethics pertained to "preserving patients' privacy and respecting human rights as well as patients’ social values and religious beliefs when offering nursing care". In the same study, nurses achieved the lowest score in relation to "consultation with hospital ethics committees when confronted with ethical challenges, delivering care outside the work environment, and introducing themselves and announcing their professional roles to patients" (23). 

However, Borhani et al. found that 81.2% of the patients were dissatisfied over the invasion of their privacy (17), and according to Dadkhah et al., most patients believed that "nurses did not respect their privacy and boundaries" (24). These findings were therefore not in line with those of the present study, which could be due to the differences between the social and educational levels of the employed manpower. It is obvious that in spite of the managers’ and nursing educators’ emphasis on adherence to professional ethics, nursing education has been impractical, and measures will need to be taken to enable and empower students and nurses to resolve this issue.

The results of this study revealed a significant relationship between gender and adherence to ethical codes related to clinical service provision and relationship with the treatment team. In both domains, women achieved higher average scores than men. In Jafari et al. study the total average score for adherence to the nursing code of ethics was higher among female students in comparison to their male counterparts as well (18). Quoting from Docket et al., Martin et al. stated that female students had a significantly higher moral reasoning score than male students at the time of initial registration and during academic education (25). Di You et al. found that in the nursing profession, women have higher moral sensitivity in comparison to men (26). Forte stated that gender differences with regard to adherence to codes of ethics and the ability to resolve ethical conflicts are caused by the different methods that men and women choose and implement to resolve conflicts (27). 

In the present study, no relationship was observed between age or work experience and the level of adherence to the code of ethics. In a study by Numminen et al., no significant relationship was observed between age or gender and the students’ understanding of ethical codes (7). In another study by Numminen et al., there was a significant relationship between age and work experience, and learning (28). A study by Auvinen et al. revealed that students who had faced real ethical problems during their education had better judgment compared to students who had not; moreover, senior students showed better judgment in comparison to freshmen (29).

The code of ethics used in the present study was published by the Ministry of Health and Medical Education in 2011 and was not new at the time of research. When we were explaining the aim of our study to the nurses, however, many of them did not even know that these codes existed, which indicates lack of a proper system of informing the nurses. Tadd et al. surveyed the nurses’ views on the content and implementation of the codes and their usage in nursing activities in six European countries. Most of the participants had little understanding of the codes. They were unfamiliar with the contents of the codes and believed they were of little clinical and practical value due to the numerous obstacles in their effective implementation (30).

Ethical codes are generally specific to the religion, culture and characteristics of a society, and therefore studies conducted in other countries are only appropriate in those contexts. Consequently, we might not be able to draw an accurate comparison between those studies and the present research. In any case, there are obstacles in applying these codes that have to be removed before we can expect their efficient implementation in clinical settings. Enactment of nursing codes of ethics requires coordination and cooperation of all nursing officials and custodians toward removing the obstacles that hinder their application as a practical charter in hospitals. 

## Conclusion

Based on our findings, nursing students had better adherence to the ethical codes on relationship with the treatment team compared to nurses. Nevertheless, they lacked the necessary know-how to adhere to the codes on clinical service provision, and were not much different from the nurses in this regard. Therefore, to improve adherence to ethical codes, certain measures will need to be taken, for instance coherent training courses must be offered on professional ethics during academic years and continuing education. Also, implementation of policies related to professional ethical codes should be enforced and supervised. Hence, it is necessary that researchers investigate the effective factors on adherence to nursing professional codes of ethics through qualitative research. Further studies need to be conducted in order to design and standardize nurses’ professional ethical practice assessment tools in accordance with the ethical codes for nurses in Iran. In addition, nursing researchers should examine the facilitators and inhibitors of observing ethical codes among nursing students and nurses, and try to enhance implementation of nursing professional ethics in clinical environments. 
